# Factors Affecting Prognosis in the Course of Pediatric Celiac Disease

**DOI:** 10.7759/cureus.32208

**Published:** 2022-12-05

**Authors:** Didem Taşkın, Alihan Sursal, Ali E Doğan, Fatih Özdener

**Affiliations:** 1 Pediatric Gastroenterology, Adana City Research and Training Hospital, Adana, TUR; 2 Neuroscience, Bahcesehir University, Istanbul, TUR; 3 Medicine, Danone, Istanbul, TUR; 4 Pharmacology, Bahcesehir University, Istanbul, TUR

**Keywords:** cross-sectional study, body mass index, growth, developmental delay, chronic disease

## Abstract

Introduction

Celiac disease (CD) is a rather frequent chronic autoimmune disease that causes impaired growth in children. The present study aims to evaluate patients’ condition after diagnosis cross-sectionally and determine the factors affecting prognosis.

Methods

Control visits were performed at the end of the 13-month intervention period. The study was designed as a single-center retrospective study and included patients diagnosed with CD. The study cohort consisted of 211 patients aged 1 to 18 years. Statistical parameters include Helicobacter positivity, Marsh classification; economic status; and body mass index (BMI) z-score, weight z-score, and height z-score to observe the difference between admission and follow-up.

Results

Treatment adherence is one of the most critical factors influencing improvement in developmental parameters during control visits (p<0.033). It was observed that the weight z-scores at the control visit deteriorated significantly with a longer duration of complaints (p=0.033). Better improvement of control visit BMI z-scores among patients with complaints compared to asymptomatic patients (p=0.036) indicate the importance of early diagnosis in asymptomatic cases. Developmental parameters of patients with CD without growth retardation (GR) show faster improvement compared to patients with GR (p<0.001). Families with good socioeconomic status can easily adapt to the diet by reaching a greater variety of gluten-free products, so anthropometric measurements are observed to be significantly higher at the control visit (p<0.002).

Conclusions

Treatment adherence is the most critical factor for improvement in CD treatment, as in all treatments. In addition, the investigation of suspected, additional disease symptoms during the follow-up of a CD patient is also of great importance for early diagnosis. The importance of early diagnosis has been emphasized in terms of anthropometric improvement in asymptomatic CD cases.

## Introduction

Celiac disease (CD) is an autoimmune disease triggered by gluten-containing foods in genetically (presence of DQ2 and/or DQ8 polymorphic human leukocyte antigen [HLA gene alleles) predisposed individuals at any age [1]. Similar to Europe and the USA, Turkey has approximately 1% prevalence of CD and is among the most common autoimmune diseases worldwide [2-4].

In CD, intestinal absorption can be impaired due to the autoimmune attack of T-cells on intestinal villi, and severe malabsorption and diarrhea are therefore one of the most common symptoms of the disease, [5] which may also lead to severe growth retardation (GR) in patients [6]. Other CD symptoms can be divided into gastrointestinal and extraintestinal manifestations. However, in some cases, the disease may progress without symptoms, making diagnosis difficult [7].

Early diagnosis, thus treatment is vital in CD, as in all diseases [8,9]. Knowing the factors causing GR in patients with CD disease beforehand of CD treatment, provides correct and early application of the nutritional intervention. The present study has investigated the effects of demographic, biochemical, histological, and anthropometric variables on the growth rate of patients with CD and the degree of improvement in anthropometric measurements via comparing data from the first admission with third month control visit. It aims to cross-sectionally evaluate patient growth after diagnosis and determine the factors affecting GR in CD.

## Materials and methods

Sample

The study was designed as a single-center retrospective study. This study was performed before the ESPGAHN and NASPGHAN recommendations for the non-biopsy diagnosis of CD thus, includes a biopsy-based diagnosis of CD for some patients. The primary inclusion criteria included the diagnosis of CD by serological analysis and/or a biopsy finding of upper gastrointestinal endoscopy. The study included a total of 211 patients between the ages of 1 and 18 years who were followed up with the diagnosis of CD in Adana City Training and Research, Hospital Pediatric Gastroenterology 1 polyclinic between September 17, 2017 and May 1, 2021. Patients with infections and other malignancies were excluded from the study. Patient files were scanned retrospectively, and sociodemographic and clinical data were recorded from the Hospital of Pediatric Gastroenterology 1 polyclinic between September 17, 2017 and May 1, 2021.

Design

The patients admitted before 2012 were diagnosed with modified Marsh classification by performing endoscopy and biopsy. Those admitted after 2012 were diagnosed with endoscopic biopsy and Marsh classification if their transglutaminase level was below 10x. In those diagnosed without biopsy, patients with tissue transglutaminase higher than 10x and positive for EMA were diagnosed according to the ESPGAHN and NASPGHAN guidelines. Biochemical variables such as Helicobacter positivity (HP); histological variables such as modified Marsh classification; anthropometric variables such as body mass index (BMI) z-score, weight z-score, height z-score; and patient information such as socioeconomic status and other diseases were analyzed. Third-month control visits were analyzed using noncoincident dates. Patient anthropometric measurements at first admission and control visit were converted into z-scores and compared statistically. Anthropometric measurements were calculated with the Cedd-Solution oxology calculation system created by the Pediatric Endocrinology and Diabetes Association [10]. Patients with GR were diagnosed in patients with < 3 percentile in both weight and height according to the published reference values for Turkish children anthropometrics [11]. Patients who only have weight or height under the 3 percentile were not included in the GR analysis. examined and divided into groups as group 1 (only height or weight below the third percentile) and group 2 (both weight and height below the third percentile). The diagnosis of HP was made by HP-specific staining in the histopathological examination of the biopsy taken from the gastric mucosa during gastroscopy.

Statistical analysis

Data were analyzed using IBM SPSS Statistics for Windows v.20.0 (IBM Corp., Armonk, NY, USA). The Shapiro-Wilk test was used to assess the normality of data distribution. For ≥ 5 and < 5 samples the chi-squared and Fisher's Exact test, respectively were used to analyze the binary categorical variables. Normally distributed data were analyzed with the Student's t-test, and non-normally distributed data were analyzed with the Mann-Whitney U test. Spearman analysis was used to compare continuous variables that did not show normal distribution. The statistical significance level was determined as p < 0.05. Spearman analysis significance level was determined as p < 0.005.

Ethical approval

Ethics committee approval was obtained by the Adana City Training and Research Hospital Ethics committee on July 1, 2021 with decision no: 1471.

## Results

A total of 211 patients aged between one and 18 years (10.6 ± 4.0) diagnosed with CD, 94.8% Turkish and 5.2% from different ethnic origins, were included in this study. Demographic information, mean growth rate, and mean anthropometric parameters at admission and control visit are presented in Table 1.

**Table 1:** Demographic data of the patients together with mean growth rates and mean anthropometric parameters at admission and control visit. SD, standard deviation; IQR, interquartile range

	Male, n (%)		Female, n (%)	
Nationality, n (%)	75 (35.5)		136 (64.5)	
Turkish	69 (92.0)		131 (96.3)	
Other Ethnicity	6 (8.0)		5 (3.7)	
Growth rate, mean ± SD (IQR), cm/3 months	1.62 ± 0.75 (0.88)		1.55 ± 0.82 (0.78)	
Growth rate, mean ± SD (IQR), cm/6 months	3.24 ± 1.49 (1.76)		3.11 ± 1.64 (1.56)	
Growth rate, mean ± SD (IQR), cm/12 months	6.36 ± 3.09 (3.53)		6.22 ± 3.27 (3.12)	
Anthropometric data	Admission	Control	Admission	Control
Weight, mean ± SD (IQR), kg	26.5 ± 14.21 (17.4)	36.6 ± 18.23 (26.0)	24.3 ± 14.17 (17.8)	33.5 ± 15.49 (23.0)
Weight z-score, mean ± SD (IQR)	-1.16 ± 1.40 (1.56)	-0.91 ± 1.15 (1.99)	-0.56 ± 2.67 (2.29)	-0.75 ± 1.34 (1.83)
Height, mean ± SD (IQR), cm	124.1 ± 24.77 (38.0)	138.3 ± 26.04 (36.0)	116.9 ± 24.27 (40.0)	133.9 ± 20.76 (35.0)
Height z-score, mean ± SD (IQR)	-1.06 ± 1.68 (1.88)	-1.01 ± 1.22 (1.70)	-0.45 ± 3.75 (2.37)	-0.96 ± 1.20 (1.92)
BMI, mean ± SD (IQR), kg/m2	16.3 ± 2.72 (3.1)	17.8 ± 3.65 (4.0)	16.4 ± 3.14 (3.5)	17.8 ± 3.69 (5.0)
BMI percentile, mean ± SD (IQR), %	29.8 ± 25.29 (38.0)	34.9 ± 31.20 (55.0)	37.3 ± 30.77 (53.0)	40.9 ± 30.30 (52.0)
BMI z-score, mean ± SD (IQR)	-0.88 ± 1.76 (1.27)	-0.52 ± 1.22 (1.82)	-0.39 ± 1.80 (1.63)	-0.50 ± 2.41 (1.48)

Additionally, the distribution of patient complaints is presented in Table 2.

**Table 2:** Complaint frequencies of the whole cohort and patients diagnosed with GR at the time of CD diagnosis. A single patient may have two complaints at the time of diagnosis. GR, growth retardation; CD, celiac disease *, the percentage is based on the group of patients diagnosed with GR

Distribution of patient complaints	Overall, n (%)	Patients diagnosed with GR, n (%)*
Abdominal pain	133 (63.0)	56 (62.9)
GR	69 (32.7)	42 (47.2)
Reflux	85 (40.3)	30 (33.7)
Nausea and vomiting	73 (34.6)	28 (34.5)
Diarrhea	76 (36.0)	25 (28.1)
Weight loss	69 (32.7)	24 (27.0)
Constipation	64 (30.3)	24 (27.0)
Joint pain	62 (29.4)	21 (23.6)
Dyspepsia	41 (19.4)	17 (19.1)
Fever	23 (10.9)	9 (10.1)

Descriptive statistics and p values of the anthropometric measurements of the entire study group and the subgroups with a significant improvement in anthropometric z-scores are presented in Table 3.

**Table 3:** Anthropometric measurements of the whole group and sub-groups with significant results. SD, standard deviation; IQR, interquartile range; GR, growth retardation

Groups	Anthropometric measurements	Admission	Control	P-value
Whole group	Weight z-score, mean ± SD (IQR)	-0.77 ± 2.32 (2.27)	-0.80 ± 1.28 (1.88)	0.096
	Heights z-score, mean ± SD (IQR)	-0.66 ± 3,19 (2.37)	-0.98 ± 1.20 (1.89)	0.363
	BMI z-score, mean ± SD (IQR)	-0.56 ± 1.79 (1.61)	-0.51 ± 2.11 (1.55)	0.153
Patients diagnosed at age >10	Weight z-score, mean ± SD (IQR)	-1.14 ± 1.23 (1.31)	-0.84 ± 1.16 (1.54)	0.018
	Heights z-score, mean ± SD (IQR)	-1.33 ± 1.42 (2.08)	-0.90 ± 1.18 (1.95)	0.002
	BMI z-score, mean ± SD (IQR)	-0.60 ± 1.06 (1.45)	-0.35 ± 1.13 (1.36)	0.034
Patients without complaints at the time of admission	Weight z-score, mean ± SD (IQR)	-0.77 ± 1.37 (2.58)	-0.31 ± 1.28 (1.91)	0.011
	Heights z-score, mean ± SD (IQR)	-0.91 ± 1.40 (2.93)	-0.48 ± 1.27 (2.01)	0.018
Marsh 3b group	Weight z-score, mean ± SD (IQR)	-1.00 ± 2.38 (1.86)	-0.74 ± 1.38 (2.24)	0.001
	Heights z-score, mean ± SD (IQR)	-1.00 ± 2.48 (2.13)	-0.92 ± 1.29 (2.13)	0.010
	BMI z-score, mean ± SD (IQR)	-0.82 ± 1.68 (1.62)	-0.34 ± 1.22 (1.68)	0.022
	Groups			
Anthropometric measurements at control visit	Patients using enteral nutrition solution	Patients not using enteral nutrition solution	P-value	
Weight z-score, mean ± SD (IQR)	-1.72 ± 0.89 (1.09)	-0.18 ± 1.12 (1.46)	< 0.001	
Heights z-score, mean ± SD (IQR)	-1.90 ± 0.92 (0.80)	-0.35 ± 0.93 (1.34)	< 0.001	
BMI z-score, mean ± SD (IQR)	-0.81 ± 1.07 (1.71)	-0.30 ± 2.57 (1.44)	< 0.001	
	Diet-compliant patients	Diet-noncompliant patients		
Weight z-score, mean ± SD (IQR)	-0.66 ± 1.28 (1.87)	-1.17 ± 1.23 (1.49)	0.007	
BMI z-score, mean ± SD (IQR)	-0.44 ± 2.39 (1.52)	-0.70 ± 1.04 (1.52)	0.033	
BMI percentile z-score, mean ± SD (IQR)	42.55 ± 31.12 (53)	-28.75 ± 27.24 (33)	0.021	
	Presence of additional disease	Absence of additional disease		
Heights z-score, mean ± SD (IQR)	-1.32 ± 1.09 (1.42)	-0.89 ± 1.21 (1.69)	0.035	
	Patients with GR as complaint	Patients without GR as complaint		
Weight z-score, mean ± SD (IQR)	-1.46 ± 1.10 (1.26)	-0.22 ± 1.14 (1.44)	< 0.001	
Heights z-score, mean ± SD (IQR)	-1.78 ± 1.00 (0.94)	-0.27 ± 0.88 (1.34)	< 0.001	
BMI z-score, mean ± SD (IQR)	-0.59 ± 1.09 (1.69)	-0.43 ± 2.71 (1.51)	< 0.001	
	Patients with complaints at admission	Patients without complaints at admission		
BMI z-score, mean ± SD (IQR)	-0.61 ± 2.21 (1.72)	-0.11 ± 1.05 (1.42)	0.036	

No significant difference was observed between the anthropometric z-scores at admission (z-scores of weight, height and BMI) versus control visit (p > 0.05 for all). Weight z-score and BMI z-score and BMI percentile of patients who complied with the given diet were significantly healthier at control visit than non-compliant patients (p = 0.007, p = 0.033, and p = 0.021, respectively). The height z-score at control visit was healthier in those without additional comorbidities such as cystic fibrosis, inflammatory bowel disease, diabetes, and thyroiditis (p = 0.035).

There was no significant difference between the anthropometric measurements of patients diagnosed within the first two years of age and those diagnosed after two years of age (p > 0.215 for z-scores of weight, height, BMI). However, separate evaluation of age groups (<2, 2-5, 5-10, >10) revealed that the anthropometric measurements of the patients diagnosed after the age of 10 were significantly higher at control visit compared to those at admission (p = 0.018, p = 0.002 and p= 0.034 for z-scores of weight, height and BMI, respectively). Although the control visit height and BMI z-scores were not found to be associated with longer durations of complaints (p = 0.076 and p = 0.064, respectively), the weight z-score was observed to deteriorate significantly at control visit (p = 0.033). Diet duration was not showed a significant correlation with antropometric measurements. 

The control visit BMI z-scores of the patients who had no complaints at the time of admission were significantly higher than those who had complaints at admission (p = 0.036). The height and weight z-scores of the patients who had no complaints at the time of admission were significantly higher at control visit (p = 0.011 and p = 0.018, respectively). HP positivity and modified Marsh classification showed no correlations with the anthropometric measurements of the patients at control visit (p > 0.250). 

Families with good socioeconomic status had significantly higher anthropometric measurements at control visit compared to families with poor socioeconomic status (p = 0.002, p = 0.016, p = 0.011 and p = 0.011, z-scores of weight, height, BMI, and BMI percentile, respectively). None of the complications, HP positivity, modified Marsh classification nor the socioeconomic status showed any correlation between growth rate (p > 0.157 for all). However mean growth rate of patients with good economic status (6.74 cm/12 months) was slightly higher than the mean growth rate of those with poor economic status (5.99 cm/12 months). 

The growth rates of patients were inversely correlated with the weight and height z-scores at admission (significance threshold p < 0.005), R = -0.178, p = 0.003 and R = -0.391, p < 0.001, respectively). There was no correlation between the BMI z-score and growth rate at admission (p = 0.278). No significant relationship (significance threshold p < 0.005) was observed between the anthropometric parameters and growth rate at control visit (p = 0.017, p = 0.112, P = 0.023 for z-scores of weight, height and BMI, respectively).

Table 4 shows the distribution of patients with and without GR in terms of HP positivity and modified Marsh classification. The comparison of the patients with GR in group 2 with patients without GR revealed no difference in HP positivity and modified Marsh classification (p = 0.282 and p = 0.573, respectively).

**Table 4:** Distribution of HP positivity and modified Marsh classification in the patients with CD who were and were not diagnosed with GR. HP, Helicobacter pylori; GR, Growth retardation *Chi-square test.

HP positivity	Patients with GR, n (% of the group)	Patients without GR, n (% of the group)
–	47 (75.8)	107 (82.3)
+	4 (6.5)	12 (9.2)
++	7 (11.3)	4 (3.1)
+++	4 (6.5)	7 (5.4)
++++	0	0
Total	62 (100)	130 (100)
P-value	0.131*	
Modified Marsh classification	Patients with GR, n (% of the group)	Patients without GR, n (% of the group)
0	0	1 (<0.1)
1	1 (<0.1)	2 (<0.1)
2	0	1 (<0.1)
3a	17 (32.7)	27 (27.0)
3b	27 (52.0)	43 (43.0)
3c	7 (13.5)	26 (26.0)
Total	52 (100)	100 (100)
P	0.673*	

## Discussion

CD is an autoimmune disease with a high worldwide prevalence (0.7%-1.4%) that occurs as a result of genetic sensitivity to gluten [12], as well as in Turkey (0.6%) [3]. Since CD symptoms can adversely affect nutrition, the presence of GR should be investigated in these cases, and, if detected, nutritional support intervention should be provided [13]. Failure to intervene in patients with childhood GR is known to increase the future risk of disease and mortality [14].

The absence of a significant overall improvement in the anthropometric z-scores stems from the fact that the number of subgroups in which no significant improvement was observed was higher than those showing improvement. When the subgroups are examined separately, factors that affect the development of patients on the CD diet, such as age at diagnosis, duration of complaints at the time of admission, and diet adherence, come to the fore. In this study, the reason why the baseline growth rate of patients who had healthier weight and height z-scores was lower than the growth rate of those with low weight and height z-scores may be due to the rapid development provided by the improvement of the villus atrophy. In other words, it can be said that they try to catch up with their peers. In addition, the reason why no significant relationship was observed between growth rate and any of the anthropometric parameters at the control visit may be due to the accumulation of the control visit values of the patients showing improvement at healthier z-score levels, closing the gap. It usually is unexpected to obtain significantly higher anthropometric z-scores in patients who do not use enteral nutrition solutions compared to those who do. It is known that patients with CD cannot reach a healthy level through enteral nutrition support alone, but the contribution of nutrition supports their development [15,16]. In our study, 92.2% of the patients using enteral nutrition solutions consisted of patients with GR as it is prescribed for GR. For this reason, despite the developmental contribution, patients using enteral nutrition solutions exhibited lower anthropometric measurements at both admission and control visits compared to those who did not.

The control visit weight z-scores, BMI z-scores, and BMI percentiles of the patients who were compliant with the prescribed diet were significantly healthier than the anthropometric measurements measured at admission, which was associated with a better improvement in the patients' developmental parameters with longer adherence to the diet. In addition, as expected, the height z-scores of patients with CD alone, that is, no other diseases such as growth hormone deficiency, diabetes, thyroiditis, and cystic fibrosis, were significantly higher than those who have any or more other diseases in addition to the CD.

It is known that accurate diagnosis of CD at the earliest possible age bears significant importance in terms of development [9-17]. Early CD diagnosis usually enables a rapid improvement in all symptoms since the elimination diet is started as early as possible [18]. When the age group at diagnosis was divided into four subgroups as <2, 2-5, 5-10, and >10 years of age, a significant improvement with the highest growth rate was observed in the control visit and admission anthropometric measurements between the patients diagnosed after the age of 10. Although improvement was observed at other ages, it was not at a level of significance. Probably this could imply associated with the onset of CD.

Increased duration of patient complaints negatively affects the control visit weight z-score because the weight reflects acute changes, whereas the height and BMI values reflect longer-term chronic changes. In addition, improved control visit BMI values among the patients without complaints show the importance of early diagnosis in asymptomatic cases, revealing the importance of investigating risk groups, namely specific syndromes, and first-degree relatives, even if they are asymptomatic.

There was no correlation between patient HP positivity and the Pathological modified Marsh classification (3a, 3b, and 3c) and anthropometric measurements and growth rates at the control visit. Although some available studies report that HP positivity causes a delay of linear growth [19-21], other publications do not associate HP positivity with GR [22-25] also HP may act as a confounding variable when it is directly proportional with the negative outcome of another disease [26] while potentially inversely related with the presence and severity of CD [27]. However, outside of undiagnosed infection, HP should not act as a confounding variable in our study as the study excludes patients with infections. Our cross-sectional study showed similar results in CD patients [9]. In this study, HP positivity was expected to make a negative difference in growth rates, but the difference was not enough to reach statistical significance. The pathological modified Marsh classification can be used for diagnostic purposes, but it is not a significant marker of the course and severity of the disease [28,29]. Therefore, as expected, no difference was detected between the modified Marsh 3a, 3b, and 3c subclasses in terms of both anthropometric measurements and growth rates. 

It is also expected that patients with good economic status have higher anthropometric measurements and growth rates, which can be explained by the rapid development facilitated by using their socioeconomic status. 

Limitations of the study

This is a retrospective observational study, thus not including control group without CD. This may cause a bias towards the prognostic effect of the nutritional intervention as well as factors affecting prognosis in CD. More studies should be performed to eliminate confounding variables.

## Conclusions

The significant improvement in developmental parameters of the patients who adhered to the diet at the control visit, especially in the weight and BMI z-scores and BMI percentile, shows that treatment adherence is the essential factor ensuring positive effects of the intervention. Some diseases such as cystic fibrosis, inflammatory bowel disease, diabetes, and thyroiditis are more common in patients with CD than in the general population and negatively affect the height z-score. Frequent monitoring and early diagnosis of suspicious symptoms in other diseases that may develop during CD follow-up are of great importance for prognosis. As expected, the developmental parameters of CD patients without GR show much faster improvement than patients with GR. Families with good socioeconomic status easily obtain gluten-free products thus, their compliance with the diet increase leading to higher anthropometric measurements at control visits. This relation of good clinical outcome with the financial situation emphasizes the requirement for overall support of these people. In these societies, other policies must be implemented that will have the main objective of effectively supporting the socially weak by solving their basic needs, such as nutrition in quantity and quality.
